# Breast Cancer Biology: The Multifaceted Roles of Mesenchymal Stem Cells

**DOI:** 10.1155/2008/425895

**Published:** 2008-12-21

**Authors:** Shyam A. Patel, Andrew C. Heinrich, Bobby Y. Reddy, Balaji Srinivas, Nicole Heidaran, Pranela Rameshwar

**Affiliations:** ^1^Graduate School of Biomedical Sciences, University of Medicine and Dentistry of New Jersey, Newark, NJ 07103, USA; ^2^Division of Hematology and Oncology, Department of Medicine, New Jersey Medical School, University of Medicine and Dentistry of New Jersey, Newark, NJ 07103, USA

## Abstract

Recent upsurge in the interest of breast cancer metastasis is partly attributed to the discovery of novel, yet unclear, mechanisms of breast cancer interaction with sites of distant metastasis such as the bone marrow microenvironment. In this review, we discuss the significance of the interactions between breast cancer cells and cells of the bone marrow. This is a subject of intense research studies aim to provide new methods of treatments and perhaps the identification of new drug targets. This review also discusses the role of inflammation and the bimodal function of the transforming growth factor-*β* signaling pathway in the process of tumorigenesis. We bring attention to future prospects in breast cancer research, including the role of microRNAs in cancer quiescence in the bone marrow and the application of microRNAs to basic science discoveries in oncology. Finally, we discuss the cancer stem cell hypothesis, which is not a new idea, but has resurged with investigative questions.

## 1. Introduction

Breast cancer, despite the subject of intense investigations, remains the most common cancer among women in the United States and the second leading cause of cancer death among this group. Each year, over 44 000 females die from breast cancer [1]. The current long-term prognosis for breast cancer is not favorable, and there remains a great deal of room for improvement in treatments, which could be derived from research [2]. Efforts to detect breast cancer early, although successful, do not guarantee survival [3]. This indicates that either more sensitive methods are needed to detect breast cancer, or it is important to understand the very early events of breast cancer biology.

The brain, bone, liver, and lungs are preferred sites of metastasis. Metastases have been reported to occur even in the absence of a primary tumor, signifying the perilous nature of this process, a subject that is revisited later in this review [4]. Metastasis to bone results in both physical and physiological imbalances such as lytic lesions and hypercalcemia [5]. Fractures, compression of the spinal cord, and reduced quality of life are the ultimate outcomes of bone metastasis [5]. Recent improvements in chemotherapy for advanced breast cancer treatment, although to be credited, have shown limited success, as chemotherapy fails to target quiescent breast cancer cells in the bone marrow [2].

The phenomenon of breast cancer cell quiescence in the marrow cavity has received attention, and several mechanisms of dormancy, as well as tertiary metastasis from the bone marrow, have been proposed [6]. Pathological triggers such as infection can promote release of both lymphocytes and cancer cells from the bone marrow. This suggests that infection can lead to tertiary metastasis of breast cancer. Although research studies have been conducted to understand the complex interplay between breast cancer cells and the bone marrow microenvironment, the mechanism remains unclear, but the significance of this disease process merits in-depth research studies.

## 2. The Cancer Cell/Bone Marrow Interface: Molecular Interactions

Molecular mechanisms of bone marrow interactions with breast cancer cells have been addressed [3]. The role of stromal cell-derived factor 1*α* (SDF-1*α*) has been given considerable attention. Bone marrow stromal cell expression of SDF-1*α* is a key consideration to understand breast cancer cell entry and integration into the bone marrow. In the region of the endosteum, SDF-1*α* expression from stromal cells interacts with the chemokine receptor 4 (CXCR4) [3]. SDF-1*α*-CXCR4 interactions have also been linked to the interface between the periphery and bone marrow cavity, thereby bringing this chemokine-receptor pair as relevant to the entry of breast cancer cells into bone marrow. Knockdown of SDF-1*α* in breast cancer cells resulted in reduced efficiency to cancer cell entry into bone marrow [3]. Following exogenous supplementation of SDF-1*α*, contact between these cells was rescued, emphasizing the critical role of SDF-1*α* in the breast cancer cell/bone marrow microenvironment [3].

**Figure 1:**
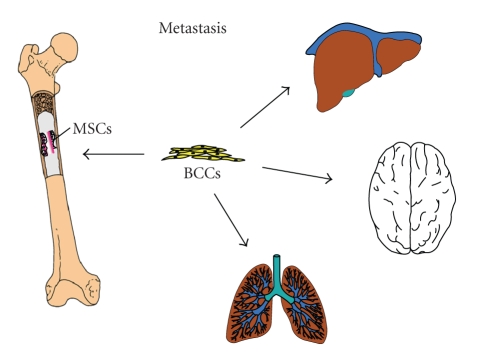
Model of bone marrow and cell migration patterns by breast cancer cells. Breast cancer cells entering the bone marrow with mesenchymal stem cells aiding the entry of the cancer cells are shown. The figure also shows the migrations of cancer cells to other distant organs. Although mesenchymal stem cells might have roles in the migration of other organs, this mechanism by which this occurs is unclear.

Since both breast cancer cells and hematopoietic stem cells express the CXCR4 receptor, it is possible that both the endogenous hematopoietic stem cells and the cancer cells can compete for “docking” to the stroma cells. During quiescence, the breast cancer cells themselves protect bone marrow destruction by decreasing the expression of SDF-1*α*, which would allow the hematopoietic stem cells to interact with stromal cells rather than the cancer cells, explained in Figure 1 [3].

We now turn out attention to the neurokinin 1 (NK1) receptor because it has been closely implicated in breast cancer interaction in the bone marrow. The interaction between SDF-1*α* and CXCR4 is regulated by the NK1 receptor, a seven-transmembrane G-protein-coupled receptor (GPCR) that has been implicated in hematological and solid malignancies [7]. The NK2 receptor has a somewhat different function and suppresses hematopoiesis [8]. The NK1 and NK2 receptors demonstrate reciprocal regulation and have opposing functions in normal cells [9].

The NK1 receptor is constitutively expressed in neural tissue but inducibly expressed in bone marrow cells and breast epithelial cells [10, 11]. The NK1 receptor interacts with peptides belonging to members of the tachykinin family. The tachykinins are encoded by the preprotachykinin-1 (*Tac1*) gene of which the major product is the undecapeptide substance P [12]. The *Tac1* gene also produces the decapeptide neurokinin A, neuropeptide K, and neuropeptide-*γ*. These products function in cell secretion and vasodilation [10]. Interactions between peptides of *Tac1* and NK1 are involved in hematopoietic regulation, depending on the interacting peptide or the signaling receptor [10]. In nontumorigenic MCF12A breast cells, activated nuclear factor-*κ*B (NF-*κ*B) has been shown to suppress *Tac1* expression level in the presence of high levels of SDF-1*α* [12]. Thus, the NK1 receptor offers a valuable pharmacologic target in diseases such as breast cancer, neuroblastoma, and hematological malignancies [7].

Recent investigations on the mechanisms of breast cancer cell metastasis to the marrow cavity have implicated a central role for *Tac1*. This gene appears to have an integral role in the molecular interaction between breast cancer cells and mesenchymal stem cells, which the cancer cells encounter upon entering the bone marrow cavity [12]. *Tac1* appears to regulate this interaction by regulating the expressions of SDF-1*α* and CXCR4 on both the cancer cells and mesenchymal stem cells. These recent findings are interesting as they could lead to future studies to define a new method of treatment by targeting the SDF-1*α*-CXCR4 interactions, and also to target the *Tac1* gene. Such treatments could be possible in the near future due to the availability of CXCR4 antagonists [12]. In summary, we propose that *Tac1* contributes to breast cancer cell metastasis and integration into bone marrow stromal compartment [9]. Additionally, *Tac1* manages the transition of breast cancer cells into a quiescent phenotype in the marrow cavity.

Recent studies suggest that particular breast cancer cell subset shows preference for the bone marrow and this preferred site could be at an early stage of the disease, perhaps prior to clinical detection [6]. At this early phase, if the cells undergo quiescence, they are supported by the bone marrow microenvironment and are likely to resist chemotherapy. It would be difficult to ignore the presence of mesenchymal stem cells at the abluminal region of blood vessels in bone marrow since they could identify avenues of treatment [6]. In addition to being able to facilitate coupling with cancer cells, mesenchymal stem cells also exert immune suppression so that the cancer cells can evade immune clearance. Thus, it is understandable why the cancer cells in bone marrow would have advantages to evade detection and protection from the innate immune system [6]. The next section selects TGF-*β* to discuss how this cytokine facilitates breast cancer cells to be established in bone marrow.

## 3. Genes Linked to Various Stages of Breast Cancer in Bone Marrow: Relevance to Resident Stem Cells

The spectrum of effects of TGF-*β* in breast cancer biology is vast, yet somewhat ambiguous. In the early stages of breast cancer growth, TGF-*β* functions as a tumor suppressor due to its antiproliferative effects [13, 14]. In later stages, TGF-*β* promotes cancer cell proliferation and metastasis, thereby functioning as an oncogene [13]. This bimodal function has been attributed to changes in the responsiveness of cancer cells to TGF-*β*. Thus, during the early stage of breast cancer development, the cells are sensitive to TGF-*β*, whereas the malignant cancer cells, as well as other carcinomas, lose this sensitivity [13]. With regards to mechanism, the TGF-*β*/SMAD signaling pathway has been attributed to the inhibition of breast cancer cell proliferation [13]. TGF-*β* also alters the microenvironment and immune responses that may provide favorable conditions for cancer maintenance [15].

The promotion of apoptosis by TGF-*β* has been linked to its interactions with the *Survivin* gene, which is a member of the inhibitor of apoptosis (IAP) family. TGF-*β* can downregulate the expression of *Survivin* at the level of gene transcription, resulting in apoptosis [14]. Evidence suggests that TGF-*β* downregulates *Survivin* via activin-like kinase 5 (ALK5) in an SMAD2- and SMAD3-dependent manner [14].

Experimental studies suggest that altered levels of *Survivin* cause changes in the receptiveness of cells to TGF-*β*, and also other cytokines responsible for inducing apoptosis [14]. These findings could be significant for combating cancer, since regulating *Survivin* levels could make breast cancer cells more susceptible to the apoptotic-inducing affect of TGF-*β*.

TGF-*β* has the ability to arrest the cell cycle progression in G1 phase via the pRb tumor suppression mechanism, thereby preventing the S phase entry and breast cancer cell proliferation [16]. However, under the regulation of *c-myc*, breast cancer cells become less susceptible to the effects of TGF-*β* [16]. Point mutations have been identified in the TGF-*β* receptor 1 (TGFR1) in breast cancer cells [13]. In contrast, mutation in the SMAD family is rarely associated with breast cancer [13]. Whereas limited expression of the type 2 TGF-*β* receptor in other cancers has been accredited to point mutation, in breast cancer it is due to the unresponsiveness to the ligand TGF-*β* [13]. Both the receptor and its intracellular signaling components are critical for regulating cell proliferation. In addition to these properties, TGF-*β* forms networks with oncogenes such as *c-myc* to modulate expression of other genes linked to tumorigenesis such as *Tac1* [13]. TGF-*β* exerts its inductive effects on *Tac1* via *c-myc* [16]. An interesting study demonstrated that TGF-*β* levels were significantly decreased in cocultures of breast cancer and bone marrow, unlike insulin-like growth factor [16]. Taken together, these extensive findings indicate that the TGF-*β* signaling pathway provides a valuable target for anticancer efforts.

Recent reports uncovered homology between the seven-transmembrane receptor, NK1, and the hematopoietic growth factor inducible neurokinin-1 type (*HGFIN*), also referred to as *nmb* [7, 17]. This finding is relevant because both NK1 and *HGFIN* have been linked to tumorigenesis, including breast cancer [7]. *HGFIN* is a type I transmembrane glycoprotein that maps to the short arm of chromosome 7 and shares structural homology to the NK1 receptor and murine Osteoactivin [7]. Based on the homology between *HGFIN* and the NK1 receptor, it would be logical for one to presume common functions by these two membrane proteins in cancer biology. However, their roles are contrasting. While NK1, in its truncated form, exerts oncogenic properties, *HGFIN* shows tumor suppressor roles [7].

*HGFIN* can interact with the major *Tac1* peptide, substance P [17]. *HGFIN* and its murine analog, Osteoactivin, exert various functions [17]. The evidence supports a tumor suppressive role of *HGFIN*. Its expression has been reported in lowly aggressive melanoma as compared to the highly aggressive melanoma [17]. The suggestion is that the role of *HGFIN* in melanoma may be attributed to its homology with the melanocyte-specific protein pMEL17 [17]. In breast cancer cells, *HGFIN* suppresses their growth and migration [17].

Similar to most tumor suppressors, *HGFIN* has also been linked to the potentiation of tumor formation but suppresses cell invasiveness. Overexpression of its homolog, Osteoactivin, has been associated with increased metastatic ability and osteolytic lesion formation in 4T1 murine breast carcinoma [5]. The transcription factor, p53, binds to multiple sites in the 5′ flanking region of *HGFIN* [16]. The limited role of *HGFIN*/*Osteoactivin* warrants future research into its link to cancer. Information on this gene has just begun as its involvement in cancer biology could be linked to the *NK* receptor gene family as well as the *Tac1* gene. The genes highlighted in this section have been associated with the biology of hematopoietic and mesenchymal stem cells [6]. Thus, these genes need to be addressed when the biology of breast cancer is studied in bone marrow with the inclusion of the two major bone marrow resident stem cells.

## 4. microRNAs: Breast Cancer Link

Recent research in the field of oncology, especially breast cancer, has focused on the concept of microRNAs (miRNAs). The technology of miRNA analyses has been employed to study the regulation of gene expression. These novel nucleotides have been implicated in cancer, myogenesis, differentiation of neurons, and stem cell renewal [18]. miRNAs are noncoding RNA molecules that are extensively processed before exerting their effects on endogenous transcripts [19]. A pri-miRNA is transcribed and then processed by Drosha and Pasha, resulting in the formation of a pre-miRNA [20]. Upon nuclear export of the pre-miRNA, it is processed in the cytosol, forming an miRNA-induced silencing complex (RISC), in which the miRNA binds to the 3′ untranslated region (UTR) of endogenous messenger RNAs [20]. Thus, miRNAs exert their effect at the translational level and are valuable in regulating gene expression. Unlike RNA interference, the RISC that is associated with miRNAs does not lead to degradation of the complex but instead leads to inhibition of endogenous message expression [20]. To date there are >500 miRNAs in the human genome [20]. The *Tac1* gene, which is linked to breast and other cancers [18], could be suppressed by translational inhibition [18]. *Tac1*, described above, regulates breast cancer cell interaction with the mesenchymal stem cells [12]. Thus, miRNAs against *Tac1* may affect quiescence of breast cancer cells in the marrow cavity [12]. Three miRNAs have been found that may bind to *Tac1*: miR-130a, miR-206, and miR-302a [17].

In addition to breast cancer, miRNAs have been implicated in the disease processes of lung cancer, colorectal cancer, and diffuse large B cell lymphoma [19]. An expression profile of miRNAs has been identified for pancreatic adenocarcinoma, including miR-221, miR-376a, miR-301 [19]. Dysregulation of miR-124 and miR-137 occurs in glioblastomas [20]. These findings represent only a fraction of our current knowledge of miRNA involvement in malignancy, yet much remains to be discovered.

In addition to their role in malignancies, miRNAs are also involved in stromal cell interaction with hematopoietic stem cells (HSCs) and the neural-hematopoietic-immunological system. Production of hematopoietic regulators including cytokines, neuropeptides, and neurotransmitters is involved in HSC functioning. Additionally, stromal cells are adaptive in their ability to respond to the aforementioned regulators [8]. Furthermore, miRNAs have been proposed to serve as the link between cancer and chronic inflammation (*see* Inflammation and Carcinogenesis), although the precise role of miRNAs in these biological processes is unclear.

## 5. Inflammation and Carcinogenesis

The bridge between carcinogenesis and chronic inflammation has been under investigation. It has been suggested that chronic inflammation can promote the formation of cancer due to increased resistance to apoptosis and increased proliferation of the affected cells [21]. Furthermore, reactive oxygen and nitrogen species which are induced by the inflammation process damage vital components of the target cell such as DNA, lipids, and protein [21]. Such damage has often contributed both directly and indirectly to malignant cell transformation. Also, the abnormal or overexpression of cytokines and other proinflammatory regulators, as well as molecules integral in intermolecular communication, promotes tumor proliferation [21]. Many cytokines commence the angiogenesis process and therefore the cancer-stromal cell communication [21].

Tumor necrosis factor *α * (TNF-*α*), a proinflammatory cytokine, has multiple roles in the development of cancer. Whereas it is potentially destructive to tumor vasculature when expressed in high concentrations, it can also facilitate tumor cell growth, including growth of breast cancer cells [21]. Mice deficient in TNF-*α* or its receptor show resistance to carcinogenesis, suggesting the oncogenic potential of TNF-*α* [21].

Interleukin-6 (IL-6) is also a proinflammatory cytokine and an acute phase reactant [22]. Specifically, IL-6 regulates the expression of antiapoptotic genes and mediates cell cycle progression [22]. Elevated IL-6 levels have been liked to the pathogenesis of cancer [22]. Treatment with anti-IL-6 results in reduced expression of the antiapoptotic protein Mcl-1, suggesting that IL-6 controls Akt-dependent survival signals [22].

The role of IL-2 in cancer cell anergy has been demonstrated. IL-2 has been shown to stimulate activation-induced cell death (AICD) as well as the proliferation of regulatory T (T_reg_) cells [23]. T_reg_ cells harbor a CD4^+^/CD25^+^ phenotype and are classified into at least two types: Foxp3^+^ T_reg_ cells utilize granzyme A to induce cell apoptosis, and Tr1/Th3 T_reg_ cells utilize granzyme B [24]. T_reg_ cells can suppress effector T cell function and autoimmunity [25]. The mechanisms of T_reg_-mediated immune suppression involve contact-dependent delivery of inhibitory signals resulting in cancer cell anergy [26].

In addition to its effects on T_reg_ cells, IL-2 has also been shown to promote the maturation of cytotoxic T lymphocytes (CTLs) to mature cells expressing CD44 and granzyme B [23]. A more complete comprehension of the consequences of these signals on CTLs is integral in the pursuit of a more effective method of adoptive immunotherapy. When activated by IL-2, CTLs have the capability to lyse tumor cells [23]. For this reason, IL-2 has been used to generate T cells to treat cancer by cell transfer techniques [23]. Additionally, IL-2 promotes CTL activation and proliferation [23]. In clinical cases, administration of IL-2 has led to cancer regression [23].

**Figure 2:**
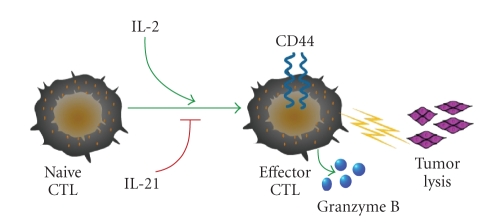
Granzyme B in cancer targeting. Granzyme B expression is increased as CTLs mature. While CD44 is moderately expressed in the early stages of CTL maturation, its expression increases during the maturation process. A mechanism by which antigen priming with IL-2 and IL-15 increases granzyme B induction is shown. In contrast, IL-21 is shown to cause opposing effect [22].

On the other end of the interleukin spectrum, IL-21 negatively regulates the effects of IL-2 (Figure 2). This demonstrates the antagonistic relationship that exists between the affects of IL-2 and IL-21 on CTLs with respect to cancer. IL-21-primed T cells were found to have the strongest antitumor response, but only in a small percentage of trials. Thus, the antitumor potential of IL-21 could be promising in future applications. Such findings are vital to adoptive immunotherapy and its implications in cancer. The potential for immune-related therapy in the treatment of cancer merits further investigation into the interplay between IL-2 and IL-21 [23].

In addition to the anticancer potential of IL-21, another member of the type I cytokine family, IL-15, has also been shown to have antiproliferative effects. IL-2 and IL-15 both promote antigen-specific cytolytic activity [23]. Despite the vast number of reports on T-cell signaling, the network and intracellular pathways triggered by T cell activation need further investigations.

The link between inflammation and stem cell in breast cancer biology is most evident in the recent studies demonstrating the anti-inflammatory effects of mesenchymal stem cells [27, 28]. Although mesenchymal stem cell therapy is not currently a target for cancer, further basic science research into their role in inflammation and cancer may provide insight into molecular processes governing the link between cancer and inflammation.

## 6. Cancer Stem Cells: New Discoveries on a Traditional Idea

The classical model for cancer is based on stochastic events that occur in a cell [1]. This model holds that a series of mutations can lead to cell transformation [1]. The development of chemotherapy agents has largely been based on this model. Recently, the concept of the cancer stem cells (CSCs) has received much attention, yet the pioneering work on the concept of the CSCs was made as early as 150 years ago. Dr. James Till helped define the term *stem cell* and, together with Dr. Ernest McCullough, coined the term *cancer stem cell* on the basis that cancer might arise from stem cells. The CSCs hypothesis is based on the identification of a unique population of stem cells in the bone marrow [29]. Among other reasons, the observation of a heterogeneous population of cells in tumors such as glioblastoma multiforme accounts for continued interest in the theory [30].

CSCs are tumorigenic multipotential cells with dysregulated self-renewal properties [31]. Upon division, one daughter cell retains stemness and the other becomes committed to a lineage [31]. The CSC fraction typically constitutes 1–5% of the tumor size [32]. They function in initiation, maintenance, growth, and metastasis of tumors [33, 34]. CSCs, like other stem cells, provide continuous source of cancer cells with limited life span, analogous to cancer progenitors. The hypothesis holds that tumors arise from developmentally arrested stem cells harboring mutations, and such characteristics promote tissue repair and organogenesis [30]. They demonstrate slow cycling and indefinite ability to renew themselves [30]. They can proliferate limitlessly and are more resistant to chemotherapy and apoptosis than somatic cancer cells [35]. The resistance of CSCs to chemotherapy and radiation is the basis for heightened interest in research endeavors in the CSCs hypothesis [30].

Evidence is gradually accumulating on the CSCs hypothesis, as numerous malignancies with stem-cell-like properties have been identified. A fraction of rat glioma cells have been found to be CD133^+^ cells with stem cell characteristics [36]. Gliomas contain 10-fold greater levels of CD133^+^ cells than normal tissue, suggesting properties of stemness in tumors [37]. CSCs have also been identified for medulloblastomas and oligodendrogliomas [29]. A recent report demonstrated that only CD133^+^ medulloblastomas and gliomas can generate tumors, with as few as 100 cells being able to recapitulate a tumor [35]. Squamous cell carcinoma of the oral cavity has been shown to be positive for the stem cell markers Oct-4, Nanog, Nestin, CD117, and CD133 [33]. These aforementioned findings are just a few pieces of evidence supporting the CSC hypothesis.

The putative breast carcinoma stem cell has received much attention. Normal stem cells of the breast can give rise to ductal epithelia, alveolar epithelia, and myoepithelia [1]. The multilineage differentiation and self-renewal properties of stem cells are evident in cells expressing CD24 and integrins *β*1 and *α*6. The breast CSCs phenotype is CD44^+^/CD24^−^/lin-, and as few as 200 cells of this phenotype can generate a tumor in NOD/SCID mice [1]. The same study demonstrated that 20 000 breast cells without this phenotype were unable to recapitulate a tumor, suggesting the significance of cancer cells with stem-like properties [1]. *HER2* overexpression has been associated with increased expression of the stem cell marker aldehyde dehydrogenase (ALDH) [32]. Furthermore, fractions of stem cell progenitors in breast tumors increase in the presence of *HER2* overexpression [32]. ALDH has been suggested as a stem cell marker that may give insight into characterization of CSCs [38].

Current chemotherapeutic treatment for cancer typically reduces tumor burden without eliminating all the cancer cells. To date, the classical cancer model has been the foundation for advances in cancer treatment. However, the high level of resistance of tumors to chemotherapy lends much support to the CSC model [1]. Multiple characteristics of CSCs account for resistance to chemotherapy and radiation, including lack of a targetable phenotype and specific oncoprotein expression, high level of *MDR1* expression, and slow cycling rate [32]. Cell-based therapy involving T cells has been proposed for the targeting of CSCs. T cells have been shown to eliminate tumors by directly targeting tumor-associated antigens [39]. For practical purposes, however, a T-cell-based regimen is difficult to administer and fails to fit into the conventional pharmacology model [39].

The significance of the CSC theory is evident from many perspectives, such as survival analyses. Triple-positivity for Oct-4, Nanog, and CD133 in oral squamous cell carcinoma renders the poorest prognosis of all squamous cell carcinoma patients [33]. These findings indicate the lethality of the CSC and merit attention to CSCs as a future target for cancer chemotherapy. By eliminating the centrally located multipotential cell characterized by dysregulation of self-renewal ability, the source of maintenance and growth of tumors would be terminated.

From a pharmacologic standpoint, methods are currently being devised to suppress CSCs. For instance, an alkylating agent used in chemotherapy for glioblastoma multiforme fails to affect CSCs that are inherently resistant [40]. The use of chloride channel antagonists, however, has shown to induce apoptosis of these CSCs [41]. The use of monoclonal antibodies to target CSCs has been proposed based on the ideas that antibodies can interfere with cancer cell signaling pathways, assist in the delivery of anticancer agents, and facilitate an immune response to tumors [34]. This approach is beneficial because it attempts to specifically target CSCs and to efficiently destroy the tumor while exerting minimal damage to healthy cells. Monoclonal antibody therapy for CSCs may thus improve prognoses [34]. Clearly, much room for pharmacological therapy against CSCs exists.

## 7. Outlook: Courses of Action to Combat Breast Cancer

The link between the chronic inflammation process and cancer growth will continue to be investigated in order to fully understand the possible functions of the various interleukins as they would relate to preventing cancer cell proliferation. Future prospects include harnessing IL-2-induced maturation of CTLs to induce apoptosis in cancer cells. Also, there is a possibility of using IL-21-primed mature CTLs as a form of external treatment as it has been shown that they have strong antitumor properties. TGF-*β* could also be a significant player in future treatments of breast cancer. Harnessing its capability to arrest the cell cycle progression would be a monumental step in the combating cancer. The anti-inflammatory properties of mesenchymal stem cells may be of value in the future of breast cancer therapy, as sites of inflammation facilitate cancer by preventing apoptosis and promoting cellular proliferation [21]. Clearly, prospects for the future in oncology research are vast.

## References

[1] Kakarala M, Wicha MS (2008). Implications of the cancer stem-cell hypothesis for breast cancer prevention and therapy. *Journal of Clinical Oncology*.

[2] Taborga M, Corcoran KE, Fernandes N, Ramkissoon SH, Rameshwar P (2007). G-coupled protein receptors and breast cancer progression: potential drug targets. *Mini Reviews in Medicinal Chemistry*.

[3] Moharita AL, Taborga M, Corcoran KE, Bryan M, Patel PS, Rameshwar P (2006). SDF-1*α* regulation in breast cancer cells contacting bone marrow stroma is critical for normal hematopoiesis. *Blood*.

[4] Katz D, Aharoni D (2004). Images in clinical medicine. Lytic lesions in breast cancer. *The New England Journal of Medicine*.

[5] Rose AAN, Pepin F, Russo C, Abou Khalil JE, Hallett M, Siegel PM (2007). Osteoactivin promotes breast cancer metastasis to bone. *Molecular Cancer Research*.

[6] Corcoran KE, Trzaska KA, Fernandes H (2008). Mesenchymal stem cells in early entry of breast cancer into bone marrow. *PLoS ONE*.

[7] Rameshwar P (2007). Implication of possible therapies targeted for the tachykinergic system with the biology of neurokinin receptors and emerging related proteins. *Recent Patents on CNS Drug Discovery*.

[8] Murthy RG, Greco SJ, Taborga M, Patel N, Rameshwar P (2008). *Tac1* regulation by RNA-binding protein and miRNA in bone marrow stroma: implication for hematopoietic activity. *Brain, Behavior, and Immunity*.

[9] Rao G, Patel PS, Idler SP (2004). Facilitating role of preprotachykinin-I gene in the integration of breast cancer cells within the stromal compartment of the bone marrow: a model of early cancer progression. *Cancer Research*.

[10] Bandari PS, Qian J, Yehia G (2003). Hematopoietic growth factor inducible neurokinin-1 type: a transmembrane protein that is similar to neurokinin 1 interacts with substance P. *Regulatory Peptides*.

[11] Patel HJ, Ramkissoon SH, Patel PS, Rameshwar P (2005). Transformation of breast cells by truncated neurokinin-1 receptor is secondary to activation by preprotachykinin-A peptides. *Proceedings of the National Academy of Sciences of the United States of America*.

[12] Corcoran KE, Rameshwar P (2007). Nuclear factor-*κ*B accounts for the repressor effects of high stromal cell-derived factor-1*α* levels on *Tac1* expression in nontumorigenic breast cells. *Molecular Cancer Research*.

[13] Kretzschmar M (2000). Transforming growth factor-*β* and breast cancer: transforming growth factor-*β*/SMAD signaling defects and cancer. *Breast Cancer Research*.

[14] Yang J, Song K, Krebs TL, Jackson MW, Danielpour D (2008). Rb/E2F4 and Smad2/3 link survivin to TGF-*β*-induced apoptosis and tumor progression. *Oncogene*.

[15] Massagué J (2008). TGF*β* in Cancer. *Cell*.

[16] Oh HS, Moharita A, Potian JG (2004). Bone marrow stroma influences transforming growth factor-*β* production in breast cancer cells to regulate c-myc activation of the preprotachykinin-I gene in breast cancer cells. *Cancer Research*.

[17] Metz RL, Patel PS, Hameed M, Bryan M, Rameshwar P (2007). Role of human *HGFIN/nmb* in breast cancer. *Breast Cancer Research*.

[18] Greco SJ, Rameshwar P (2007). MicroRNAs regulate synthesis of the neurotransmitter substance P in human mesenchymal stem cell-derived neuronal cells. *Proceedings of the National Academy of Sciences of the United States of America*.

[19] Lee EJ, Gusev Y, Jiang J (2007). Expression profiling identifies microRNA signature in pancreatic cancer. *International Journal of Cancer*.

[20] Papagiannakopoulos T, Kosik KS (2008). MicroRNAs: regulators of oncogenesis and stemness. *BMC Medicine*.

[21] Kundu JK, Surh Y-J (2008). Inflammation: gearing the journey to cancer. *Mutation Research/Reviews in Mutation Research*.

[22] Wei L-H, Kuo M-L, Chen C-A (2001). The anti-apoptotic role of interleukin-6 in human cervical cancer is mediated by up-regulation of Mcl-1 through a PI 3-K/Akt pathway. *Oncogene*.

[23] Hinrichs CS, Spolski R, Paulos CM (2008). IL-2 and IL-21 confer opposing differentiation programs to CD8^+^ T cells for adoptive immunotherapy. *Blood*.

[24] Korten S, Badusche M, Büttner DW, Hoerauf A, Brattig N, Fleischer B (2008). Natural death of adult *Onchocerca volvulus* and filaricidal effects of doxycycline induce local FOXP3+/CD4+ regulatory T cells and granzyme expression. *Microbes and Infection*.

[25] Fernandez MA, Puttur FK, Wang YM, Howden W, Alexander SI, Jones CA (2008). T regulatory cells contribute to the attenuated primary CD8^+^ and CD4^+^ T cell responses to herpes simplex virus type 2 in neonatal mice. *The Journal of Immunology*.

[26] Vasir B, Wu Z, Crawford K (2008). Fusions of dendritic cells with breast carcinoma stimulate the expansion of regulatory T cells while concomitant exposure to IL-12, CpG oligodeoxynucleotides, and anti-CD3/CD28 promotes the expansion of activated tumor reactive cells. *The Journal of Immunology*.

[27] Leal A, Ichim TE, Marleau AM, Lara F, Kaushal S, Riordan NH (2008). Immune effects of mesenchymal stem cells: implications for Charcot-Marie-Tooth disease. *Cellular Immunology*.

[28] Hagiwara M, Shen B, Chao L, Chao J (2008). Kallikrein-modified mesenchymal stem cell implantation provides enhanced protection against acute ischemic kidney injury by inhibiting apoptosis and inflammation. *Human Gene Therapy*.

[29] Wong JF (2007). Probing the biology of cancer stem cells: AACR sheds light on the microenvironment to better target these cells and their pathways. *Genetic Engineering & Biotechnology News*.

[30] Stiles CD, Rowitch DH (2008). Glioma stem cells: a midterm exam. *Neuron*.

[31] Hambardzumyan D, Squatrito M, Carbajal E, Holland EC (2008). Glioma formation, cancer stem cells, and Akt signaling. *Stem Cell Reviews*.

[32] Korkaya H, Paulson A, Iovino F, Wicha MS (2008). HER2 regulates the mammary stem/progenitor cell population driving tumorigenesis and invasion. *Oncogene*.

[33] Chiou S-H, Yu C-C, Huang C-Y (2008). Positive correlations of Oct-4 and Nanog in oral cancer stem-like cells and high-grade oral squamous cell carcinoma. *Clinical Cancer Research*.

[34] Okamoto OK, Perez JF (2008). Targeting cancer stem cells with monoclonal antibodies: a new perspective in cancer therapy and diagnosis. *Expert Review of Molecular Diagnostics*.

[35] Annabi B, Rojas-Sutterlin S, Laflamme C (2008). Tumor environment dictates medulloblastoma cancer stem cell expression and invasive phenotype. *Molecular Cancer Research*.

[36] Shen G, Shen F, Shi Z (2008). Identification of cancer stem-like cells in the C6 glioma cell line and the limitation of current identification methods. *In Vitro Cellular & Developmental Biology - Animal*.

[37] Shervington A, Lu C (2008). Expression of multidrug resistance genes in normal and cancer stem cells. *Cancer Investigation*.

[38] Douville J, Beaulieu R, Balicki D ALDH1 as a functional marker of cancer stem and progenitor cells.

[39] Brenner MK (2008). Developing T-cell therapies for cancer in an academic setting. *Advances in Experimental Medicine and Biology*.

[40] Beier D, Röhrl S, Pillai DR (2008). Temozolomide preferentially depletes cancer stem cells in glioblastoma. *Cancer research*.

[41] Kang M-K, Kang S-K (2008). Pharmacologic blockade of chloride channel synergistically enhances apoptosis of chemotherapeutic drug-resistant cancer stem cells. *Biochemical and Biophysical Research Communications*.

